# Looking Forward

**Published:** 1889-09

**Authors:** S. H. Preston


### HALL'S JOURNAL OF HEALTH

TRUTH DEMANDS NO SACRIFICE: ERROR CAN MAKE NONE.

VOL. 36. SEPTEMBER, 1889. No. 9.

### LOOKING FORWARD.

BY S. H. PRESTON.

No. 1.

The greatest glorification ever given by any people, has just been ours over our growth for a hundred years. Our rapid increase in population is unprecedented in the history of the race. During the century it has been more than sixteen times the original number. Not only have our native numbers doubled faster than any other portion of mankind, but they have been augmented many millions by immigration.

To be sure, we occupy a vast and fertile section of the earth's surface. But it has been fastly filling up within the last few decades. Once we could wisely welcome the hungry hordes of the Old World.

"Across the wide Atlantic's breast
The steamers bear them on;
The laden trains are rushing west
To greet the setting sun;

Along the broad Missouri slope
The living tide will pour;
So let them come, we've ample room
For many millions more."

But now the millions have come, and we have no more room for the rest of the world's starved out and shiftless surplus. The stupendous steamships that speed along the Atlantic like lines of ocean railway, have been weekly dumping upon our shores an undesirable population equal to a good sized city. Our factories and poor houses and prisons have been filled from foreign ports. It is high time a check were set to the squalor and crime being shifted to our shores from other continents. Sixty-five hundred steerage possengers have been landed at Castle Garden alone in a single day. In 1877 the number of immigrants to all our ports was less than 150,000. In 1880, the number reached 458,000. During the next eleven years of this century, it is probable that, unless Congress interposes some restriction, five or six millions more will reach our Republic, or half as many as arrived during the whole sixty years from 1820 to 1880 ; and that the closing census of the century will give us a population of 100,000,000.

The surprising rate at which we are filling up our territory is shown from the fact that in three quarters of a century we have gained 10,000, 000 more inhabitants than great Britain has in a century and three quarters. While the population of England has increased four-fold ours has increased ten-fold. And yet English economists are advocating emigration as the only escape from the evils of over population. And good reason. Statistics show that there is now one pauper in every thirty-three of a population that annually adds a quarter of a million to its numbers.

Now, suppose we got a slate and pencil. Population increases in a geometrical ratio, doubling every twenty-five years, or four times in a century. Let 1 express present population. At the end of twenty-five years, it will be two ; in fifty years it will have doubled again, 4 ; in another twenty-five years, 8 ; and at the end of the century, 16. Apply this calculation to the natural increase of a single pair of human beings. In fifteen hundred years it would amount to more than thirty-five thousand one hundred and eighty-four times as many as the present population of the world. To reckon the multiplication of man since the time of Moses, had it continued unchecked, would require the algebra of an archangel with boundless space for a balance sheet.

Say that for the future our population will double but three times in a century, and put it at present at only fifty millions ; two hundred years, 3,200,000,000 ; three hundred years, 25,600,000,000. And now we need a larger slate. Set apart so much space for each person, and see how long before all the standing room between the oceans would be filled. In five centuries there would be thirty-two thousand seven hundred and sixty-eight times as many as at first. And now where will we get a slate big enough to express the population of this country, even at the this reduced rate of increase, should it continue till A. D. 2900 ?

Of course, population could nowhere on earth long continue even this low rate of increase for lack of the means of subsistence. For the soil, under the most favorable conditions, cannot be made to increase its food supply faster than an arithmetical ratio, while population doubles in a geometrical ratio. So that the increase of food would fail to keep pace with that of population at the end of twenty-five years. Suppose it possible for the population of the entire earth to maintain this rate of multiplication for a single century, that is, double three times. It would exceed by more than three times the utmost possible increase of the food supply. When the population reached 500,000 millions there would be food for only 10,000 millions, or a fiftieth part of the number.

There is a limit to the food productions of the earth. There is no limit to population save that fixed by famine. And at the present rate of human increase there is no place on this planet but must, in the course of time, become incapable of sustaining its inhabitants. However plentiful food may be in any land, it is a limited supply, and population will outstrip it in time. And unless some process be discovered by which provisions may be produced with as much facility as families, the whole world will, at some period, become over-populated up to the starvation check.

Here in America we have multiplied rapidly because there has been a demand for men. The production of men, like that of any other commodity, is regulated by the law of demand and supply. Our country contained prodigious possibilities of prosperity We needed numbers to develope our infant industries, to turn our wildernesses into wheat fields and to wrest wealth from the unfenced farms of our wonderful West. Ours was a New World. When it becomes as populous as the Old it will be as full of poverty and distress.

There are people so sanguine as to suppose there is room upon our great prairies for the whole human family, and that sufficient food for all could be raised in the vast valley of the Mississippi, but stupendous as our resources are, they have a limit, and we are nearing that limit every day. The crop bearing capacities of our tracts of untilled land can be taxed to just a certain extent, and beyond that there is no Yankee trick of agriculture by which another grain can be obtained.

An Iowa statistician states that "The darkest cloud hanging over the future of our country is over-population." He further declares, "If we could stop immigration, our natural increase from our present numbers would fill our territory all too full in our next hundred years." Political economists and far-seeing statesmen look serious as they remove their rose-colored spectacles and peer into futurity. For flood and famine must work faster than they have to stay the human swarms that seek but a bare subsistence.

Already the number of workers far exceeds the demand for work. Every trade and line of labor is over-full, and hungry hundreds stand ready to squeeze into any vacant spot upon the treadmill of toil. An army of a million idle workmen are beginning to display their threatening teeth to the face of uncompassionate capital, and manufacturing towns are torn by the strife of strikers with those fighting for their places at any wages.

The lot of our working millions is quite miserable enough, leaving out of question the million for whom there is no work. Yet our toilers are kings compared with their British brothers, whose fare is far the best of any in trans-Atlantic lands. Their condition there, in the most comfortable country of Continental Europe, is such that a stout spadesman will work and support himself for $150 per year, sewing women for 1½ cents an hour and furnish their own thread and needles, and a class of dock laborers in London, numbering 20,000, are glad to get $1.50 per week. It is said that these laborers present a pitiful sight as they throng the docks fighting among themselves for tickets admitting them to one day's work, only a quarter of them being able to procure employment at even that pittance. And there are women who house themselves in stables during the winter, making lace for a living by the warmth of cows, and children who become blind there in those cattle quarters working by the light of lamps. And there are miners who chew coal to cheat hunger, fishermen who feed on grass when the fishery fails, and hunters of work who faint from hunger under hedge rows. While thousands of our bread winners have but half a loaf, tens of thousands of their European brethren are absolutely breadless.

We are the most prosperous people on the globe, and poverty is assuming an appalling attitude with us. The paupers constantly cared for by the public charities of New York City, number twice the force of the fire and police departments combined, and equal half the standing army of the United States. Besides numerous religious and private societies for the aid of the poor, the city contains three hundred and twenty public charitable institutions, which annually expend nearly nine millions of dollars ; and these are found insufficient to supply the daily increasing demands of the destitute. The city also cares for three thousand infants in its foundling hospitals.

Poverty is the most grievious curse which clouds our country to-day. It pack humanity into unhealthy slums and hovels, chains childhood and trembling age to galleys of drudgery, shuts out the sunshine of joy, and shortens the span of life. An honest, hopeless poverty is becoming more and more the common condition of mankind. Wages are lowering, laborers are doubling, and millions of the great industrial masses are but a step from destitution, many of them liable at the next deal of the commercial cards to become charges on

> "Organized charity, scrimped and iced,
> In the name of a cautious, statistical Christ."

But we are told that the world is gainining wonderfully in wealth with each generation. And according to statistics it secms to be so. We will look into that later on. Maybe we have been adopting false standards of wealth. And statistics also show that pauperism has been outstripping population for a century. Woful, wide-spread, deplorable destitution is a fact that confronts us everywhere. With all this boasted progress in prosperity, are the masses of mankind better provided with the comforts of life ?

Lord Brougham once declared in the British Parliament that a few generations ago the workingmen in all civilized conntries enjoyed the blessings of a home, but that majorities of them were in a state but little better than beggary and serfdom. It matters not how much the wealth of the world increases, so long as the poorer classes everywhere are multiplying their numbers and gradually growing poorer. The startling statement is made in a book entitled "Begging For Bread" that eight hundred millions of the earth's population are landless and homeless.

